# XIAP Regulates Cytosol-Specific Innate Immunity to *Listeria* Infection

**DOI:** 10.1371/journal.ppat.1000142

**Published:** 2008-08-29

**Authors:** Laura D. Bauler, Colin S. Duckett, Mary X. D. O'Riordan

**Affiliations:** 1 Department of Microbiology and Immunology, University of Michigan Medical School, Ann Arbor, Michigan, United States of America; 2 Department of Pathology, University of Michigan Medical School, Ann Arbor, Michigan, United States of America; Institut Pasteur, France

## Abstract

The inhibitor of apoptosis protein (IAP) family has been implicated in immune regulation, but the mechanisms by which IAP proteins contribute to immunity are incompletely understood. We show here that X-linked IAP (XIAP) is required for innate immune control of *Listeria monocytogenes* infection. Mice deficient in XIAP had a higher bacterial burden 48 h after infection than wild-type littermates, and exhibited substantially decreased survival. XIAP enhanced NF-κB activation upon *L. monocytogenes* infection of activated macrophages, and prolonged phosphorylation of Jun N-terminal kinase (JNK) specifically in response to cytosolic bacteria. Additionally, XIAP promoted maximal production of pro-inflammatory cytokines upon bacterial infection *in vitro* or *in vivo*, or in response to combined treatment with NOD2 and TLR2 ligands. Together, our data suggest that XIAP regulates innate immune responses to *L. monocytogenes* infection by potentiating synergy between Toll-like receptors (TLRs) and Nod-like receptors (NLRs) through activation of JNK- and NF-κB–dependent signaling.

## Author Summary

During a bacterial infection, the innate immune response plays two critical roles: controlling early bacterial replication and stimulating the adaptive immune response to clear infection. Host recognition of bacterial components occurs through pathogen sensors at the cell surface or within the host cell cytosol. Inhibitor of apoptosis proteins (IAPs) have been recently implicated in immune regulation, but how IAPs contribute to immunity is incompletely understood. Here, we show that X-linked IAP (XIAP) protects against infection by the cytosolic bacterial pathogen, *Listeria monocytogenes*, which causes severe disease in neonates and immunocompromised individuals. We found that XIAP enhanced MAP kinase signaling in *L. monocytogenes* infected macrophages, a key innate immune effector cell. Additionally, XIAP enabled synergy between cell surface and cytosolic bacterial sensors, promoting increased gene expression of proinflammatory cytokines. Our findings suggest that IAPs are integral regulators of innate immune signaling, coordinating extracellular and intracellular responses against microbial components to control bacterial infection.

## Introduction

The inhibitor of apoptosis (IAP) family of proteins plays a key role in cellular signaling, such as apoptosis, by binding to pro-apoptotic proteins, interrupting the intrinsic programmed cell death pathway and activating anti-apoptotic mechanisms [1]–[3]. In addition to modulating apoptosis, recent genetic studies have revealed that a *Drosophila* IAP protein, *diap2*, acts as a regulator of anti-microbial immunity [4]–[7]. Innate immune signaling pathways are well conserved from *Drosophila* to humans, suggesting that IAP proteins may also play a role in mammalian innate immunity [8]. This hypothesis is consistent with a study demonstrating that cIAP2 exacerbates endotoxic shock in mice by controlling macrophage apoptosis [9]. Furthermore, a cohort of patients with X-linked lymphoproliferative syndrome (XLP) were found to have mutations in the gene encoding XIAP, resulting in a primary immunodeficiency [10]. XIAP, also known as BIRC4 and hILP, contains three baculoviral IAP repeat (BIR) domains, the characteristic protein-protein interaction domain of the IAP family [11]. XIAP also has a carboxy-terminal RING domain with E3 ubiquitin ligase activity that directs proteasomal degradation of target proteins [12]. Multiple signaling pathways can be modulated by XIAP, including NF-κB, MAP kinase and TGFβ signaling [13]–[16]. Moreover, XIAP can integrate cellular responses to diverse stimuli by interacting directly with ligands such as copper to regulate copper homeostasis [17]. XIAP has been predominantly characterized as an inhibitor of apoptosis, and interacts with many known mediators of programmed cell death, such as JNK, TAK1, TAB1, TRAF6, and caspases-3, -7, and -9 [3],[13],[18],[19]. However, XIAP-deficient mice do not appear to have striking defects in apoptosis, thus the role of XIAP *in vivo* is not yet clearly understood [20].

The innate immune response protects host organisms against invading pathogens prior to the onset of adaptive immunity. Pathogens stimulate innate immune signaling through pattern recognition receptors (PRR), which recognize well-conserved pathogen-associated molecular patterns (PAMPs) [21]. PAMPs are detected at the host membrane by TLRs, and in the cytosol by the NLR and the RIG-I-like helicase (RLH) sensors [22],[23]. Stimulation of either extracellular or intracellular PRR can result in activation of NF-κB and MAP kinase signaling pathways, leading to production of inflammatory mediators such as cytokines and costimulatory molecules [24]. Activation of TLRs and NLRs together can induce synergy between the signaling pathways, resulting in enhanced activation of innate and adaptive immunity [25],[26]. *Listeria monocytogenes* is a cytosolic bacterial pathogen used extensively to probe aspects of innate and adaptive immunity [27]. *L. monocytogenes* is recognized by TLRs expressed on the surface of phagocytes [27]. After phagocytic uptake, *L. monocytogenes* escapes from host vacuoles by secreting a pore-forming toxin, listeriolysin O (LLO) [28]. Once in the cytosol, *L. monocytogenes* can trigger oligomerization and signaling by NOD1 and other NLRs [29]. Here we show that XIAP plays a protective role during infection by *L. monocytogenes*. We present evidence that amplifying JNK activation and subsequent pro-inflammatory cytokine production in response to cytosolic bacteria is one mechanism by which XIAP modulates innate immunity.

## Results

### XIAP regulates innate immunity to *L. monocytogenes*

**Figure 1:**
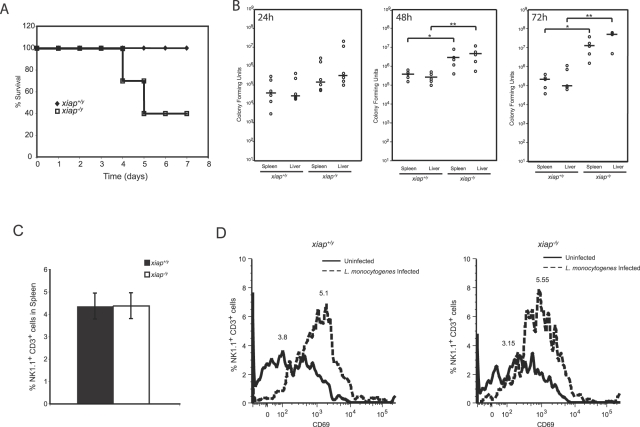
XIAP protects against *L. monocytogenes* infection during the innate immune response. (A) Survival curve of *L. monocytogenes* in *xiap^+/y^* and *xiap^−/y^* mice. Mice were injected with 1×10^5^ *L. monocytogenes* intraperitoneally, and survival was monitored daily (n = 10 animals per group). (B) CFUs isolated from the liver or spleen of mice infected with 5×10^5^ *L. monocytogenes* intraperitoneally at 24, 48, and 72 hpi. Each point represents one animal. Mean CFUs is indicated by a horizontal line. *p≤0.05; **p≤0.005. (C) Flow cytometry analysis of NK1.1^+^CD3^+^ NKTCs in the spleens of uninfected *xiap^+/y^* and *xiap^−/y^* animals (error bars represent SD). (D) Activation of NKTCs during *L. monocytogenes* infection. Splenocytes were harvested from infected animals at 48 hpi, and stained with NK1.1-biotin, CD3-FITC, and CD69-PE fluorescent-coupled antibodies for flow cytometry analysis. Results are representative of three independent experiments (n = 9 animals).

We first tested the hypothesis that XIAP contributed to anti-microbial immunity by infecting *xiap^+/y^* and *xiap^−/y^* mice with 1×10^5^ *L. monocytogenes* and determining survival over time (Figure 1A). At 7 dpi (days post infection), 60% of the XIAP-deficient mice had succumbed to infection, whereas all wild-type mice survived. Similarly, at higher doses of *L. monocytogenes* more *xiap^−/y^* than *xiap^+/y^* mice succumbed to infection, although some *xiap^+/y^* mice also became moribund (unpublished data). Depending upon the inoculum, morbidity and mortality of *xiap^−/y^* animals occurred between 2 and 5 dpi, prior to peak development of adaptive immunity, suggesting that XIAP had a protective effect during the innate response to bacterial infection. To better define the role of XIAP during innate immunity to intracellular bacterial infection, we infected wild-type and XIAP-deficient mice intraperitoneally with 5×10^5^ *L. monocytogenes*, and harvested spleen and liver to enumerate bacterial burden at 24, 28 and 72 hpi (Figure 1B). By 48 h, *xiap^−/y^* mice had approximately 10-fold more *L. monocytogenes* in liver and spleen at 48 hpi compared to the *xiap^+/y^* mice, consistent with our observation of their decreased survival. At 72 hpi, the difference between the *xiap^+/y^* mice and the *xiap^−/y^* was even more pronounced, with the *xiap^−/y^* mice supporting 100-fold greater bacterial numbers. These results indicate that XIAP mediates innate resistance to *L. monocytogenes* infection.

Mutations in XIAP have been associated with the human immunodeficiency syndrome, XLP [10]. One feature associated with this disease is an abnormally low number of natural killer T-cells (NKTCs), although it is not yet clear how much this phenotype contributes to immunodeficiency. To determine if mice lacking XIAP exhibit a similar phenotype to XLP patients, we quantitated the percentage of NKTCs in the spleen of *xiap^+/y^* and *xiap^−/y^* mice (Figure 1C). No significant difference in the number of splenic NKTCs was observed between *xiap^+/y^* and *xiap^−/y^* mice, indicating that survival of NKTCs in uninfected mice is not affected by a deficiency in XIAP, consistent with a previous report [10]. To determine if NKTC survival or activation was dependent on XIAP during *L. monocytogenes* infection, we infected animals and determined the number of splenic NK1.1^+^CD3^+^ NKTCs that expressed CD69, a marker of activation (Figure 1D). We observed similar numbers of activated NKTCs in *xiap^+/y^* and *xiap^−/y^* mice. These data suggest that XIAP does not play an important role in NKTC survival or activation in a murine model of listeriosis.

**Figure 2:**
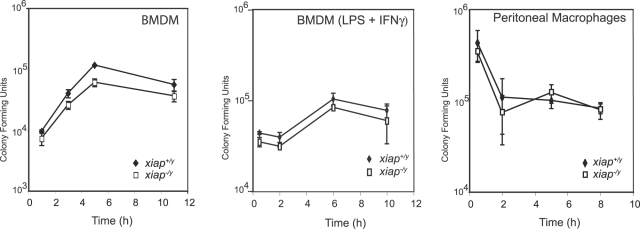
XIAP does not restrict growth of *L. monocytogenes* in primary macrophages *ex vivo*. Intracellular growth of *L. monocytogenes* in unactivated, activated, and peritoneal macrophages. Unactivated macrophages were infected at an MOI of 1. Activated macrophages were stimulated overnight with 10 ng/ml LPS and 10 ng/ml interferon-γ. Activated and peritoneal macrophages were infected with an MOI of 10 (error bars represent SD).

We then tested the role of XIAP during infection of primary macrophages, an innate immune effector cell and a well-characterized host for *L. monocytogenes* replication. We infected unactivated bone marrow derived macrophages (BMDMs), BMDMs activated with LPS and IFNγ or peritoneal macrophages with *L. monocytogenes* and measured intracellular bacterial growth over time (Figure 2). All types of *xiap^+/y^* and *xiap^−/y^* macrophages controlled *L. monocytogenes* infection equally well. We conclude from these data that XIAP does not contribute directly to restriction of *L. monocytogenes* growth in macrophages, even though XIAP-deficient mice exhibited an increased bacterial burden compared to wild-type mice. Taken together, our results demonstrate that XIAP is required for a protective immune response to *L. monocytogenes* infection *in vivo*.

### Translocation of NF-κB in response to *L. monocytogenes* is enhanced by XIAP

**Figure 3:**
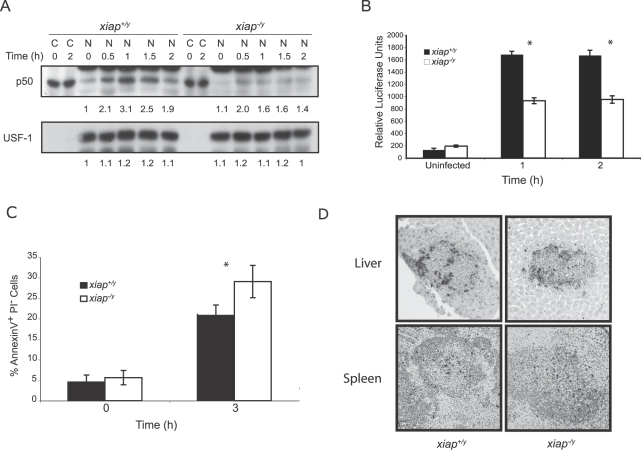
XIAP enhances NF-κB translocation during *L. monocytogenes* infection. (A) Nuclear translocation of p50 in *xiap^+/y^* and *xiap^−/y^* activated BMDM in response to wild-type *L. monocytogenes* infection. Cells were activated with 10 ng/ml LPS and 10 ng/ml interferon-γ overnight, and infected at an MOI of 10 for 30 min. Upon lysis, the nuclear fraction (N) was separated by centrifugation from the cytosolic fraction (C). Data are representative of at least 3 independent experiments. (B) DNA binding activity of p50/p65 as measured by ELISA. Nuclear extracts from *xiap^+/y^* and *xiap^−/y^* activated BMDM that were uninfected or infected with wild-type *L. monocytogenes* were added to 96-well dishes coated with a canonical NF-κB consensus DNA binding sequence, followed by detection with a p65-specific antibody. Results are representative of at least 3 independent experiments (error bars represent SD). (C) Flow cytometry analysis of apoptosis in activated BMDM infected with *L. monocytogenes* at 3 hpi. Macrophages were stained at the indicated times post infection with Annexin V-FITC and propidium iodide. Results are representative of at least 3 independent experiments (error bars represent SD of macrophages from 3 mice). (D) TUNEL staining of histological sections of livers and spleens from *xiap^+/y^* and *xiap^−/y^* mice infected with *L. monocytogenes* for 48 h (n = 3 animals/genotype). Ten sections per animal were examined.

XIAP can activate NF-κB–dependent transcription in response to apoptotic stimuli [14]. In addition to regulating apoptosis, the canonical NF-κB p50/p65 heterodimer has a well-established role in proinflammatory cytokine transcription stimulated by TLR and NLR signaling [21]. Expression profiling of unactivated macrophages infected with *L. monocytogenes* did not reveal reproducible differences between wild-type and XIAP-deficient macrophages (unpublished data). We then reasoned that activated macrophages might be a more relevant environment for studying XIAP function. We therefore investigated whether XIAP regulated NFκB-dependent processes during *L. monocytogenes* infection in activated macrophages by measuring translocation of p50 to the nuclear compartment. Activated BMDM were infected with wild-type *L. monocytogenes*, and translocation of the p50 subunit of NF-κB was analyzed by immunoblot (Figure 3A). As early as 0.5 hpi, p50 was detected in the nuclear fraction of both *xiap^+/y^* and *xiap^−/y^* cells; however, in the presence of XIAP there was substantially more p50 in the nuclear fraction over time. We also measured DNA binding activity of the p65 subunit of the p50/p65 heterodimer in the nuclear fraction of uninfected and *L. monocytogenes* infected activated macrophages (Figure 3B). At 1 and 2 hpi, infected *xiap^+/y^* macrophage nuclear lysates contained significantly more NF-κB DNA binding activity than infected *xiap^−/y^* nuclear lysates, suggesting that XIAP might enhance signaling of NF-κB–dependent pathways stimulated by bacterial infection.

In some contexts, XIAP-dependent NF-κB activation can protect against apoptotic stimuli; therefore we tested if XIAP modulated apoptosis during *L. monocytogenes* infection. We first examined apoptosis in activated macrophages during *L. monocytogenes* infection by flow cytometry of infected cells using Annexin V (AnnV), an indicator of apoptosis (Figure 3C). A modest but reproducible increase in apoptosis was observed by 3 hpi in XIAP-deficient macrophages compared to wild-type macrophages, which remained consistent throughout infection (Figure S1A). We also examined apoptosis in infected liver and spleen at sites of *L. monocytogenes* replication 48 hpi by performing TUNEL staining (Figure 3D). Although the extent of apoptosis at foci of infection were heterogeneous, there did not appear to be any notable difference in the number or distribution of apoptotic cells per focus in *xiap^+/y^* compared to *xiap^−/y^* livers or spleens. We did not observe any XIAP-dependent difference in the numbers of AnnV^+^ T or B cells present in the spleens of mice at 48 hpi (Figure S1B). In addition, caspase-3 cleavage in infected activated macrophages was not significantly altered (unpublished data). While the infected *xiap^−/y^* macrophages exhibited a modest increase in cell death, we found no striking evidence for regulation of apoptosis by XIAP in the context of *L. monocytogenes* infection *in vivo*. Thus, XIAP regulates NF-κB activation during *L. monocytogenes* infection, but may enhance innate immunity by modulating cellular responses other than apoptosis in infected macrophages.

### XIAP modulates JNK activation in response to cytosolic *L. monocytogenes*

**Figure 4:**
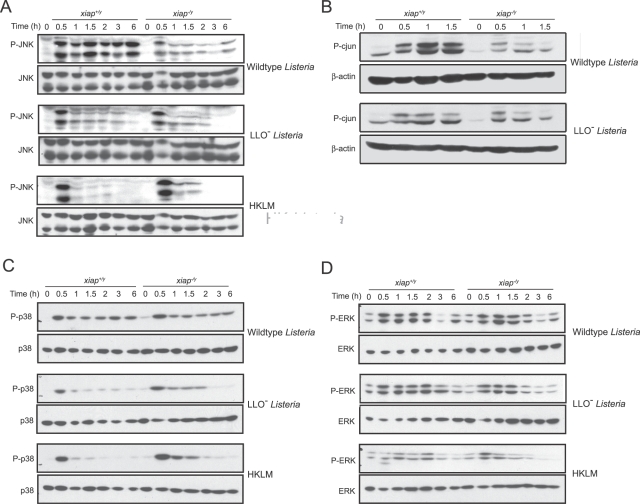
XIAP prolongs JNK signaling in response to cytosolic *L. monocytogenes*. Immunoblot of lysates from *xiap^+/y^* and *xiap^−/y^* activated BMDM that were uninfected or infected with wild-type, or LLO^−^ *L. monocytogenes* or HKLM. Cells were activated overnight with 10 ng/ml LPS and 10 ng/ml interferon-γ, followed by infection at an MOI of 10 for 30 min. Cells were lysed and subjected to immunoblot analysis using anti-JNK, anti-phospho-JNK, anti-phospho-c-jun, anti-c-jun, anti-phospho-ERK, anti-ERK-1, anti-phospho-p38, and anti-p38 antibodies. Data are representative of at least 3 independent experiments. (A) JNK phosphorylation. (B) c-jun phosphorylation. (C) p38 phosphorylation. (D) ERK phosphorylation. Quantitation of blots can be found in Figure S2.

In addition to NF-κB activation, TLR and NLR sensing of microbial infection stimulate MAP kinase phosphorylation, leading to activation [30]. Previous reports suggested that XIAP can promote JNK phosphorylation via interaction with TAB1 and the MAP3K, TAK1 [14],[16],[31]. To determine if XIAP affected JNK phosphorylation during *L. monocytogenes* infection, we performed immunoblot analysis of infected lysates from *xiap^+/y^* and *xiap^−/y^* activated macrophages using a phospho-JNK specific antibody (Figures 4A and S2A). Upon infection with wild-type *L. monocytogenes*, JNK phosphorylation occurred as early as 0.5 hpi in both *xiap^+/y^* and *xiap^−/y^* cells. In the *xiap^−/y^* macrophages, JNK phosphorylation peaked at 0.5 hpi. However, in the presence of XIAP, enhanced JNK activation was prolonged up to 6 h. This suggests that XIAP augments JNK signaling during wild-type *L. monocytogenes* infection. To determine the contribution of XIAP to cytosol-specific signaling, we compared wild-type *L. monocytogenes* infection with a strain deficient in LLO or heat-killed *L. monocytogenes* (HKLM), which both remain trapped in the vacuole. The LLO^−^ bacteria and HKLM induced JNK phosphorylation at 0.5 hpi similarly to infection by wild-type bacteria, suggesting that this early JNK phosphorylation was linked to signaling from the vacuole, most likely through TLRs. However, JNK phosphorylation in response to vacuolar bacteria quickly diminished after 30 min, in contrast to the extended XIAP-dependent JNK activation observed during wild-type bacterial infection. To confirm that enhanced JNK phosphorylation in *xiap^+/y^* activated macrophages resulted in downstream signaling, we examined phosphorylation of c-jun, a target of JNK, by immunoblot (Figures 4B and S2B) [32]. Upon infection by wild-type *L. monocytogenes*, c-jun phosphorylation was prolonged in *xiap^+/y^* but not *xiap^−/y^* cells, similarly to JNK phosphorylation. Moreover, activation of c-jun upon infection by LLO^−^ bacteria was considerably decreased compared to wild-type bacteria. To determine if XIAP also stimulated activation of other MAP kinase family members, we analyzed phosphorylation of p38 and ERK by immunoblot of infected macrophage lysates (Figures 4C, 4D, S2C, and S2D). ERK1 and ERK2 were phosphorylated equivalently in *xiap^+/y^* and *xiap^−/y^* macrophages in response to infection by all *L. monocytogenes* strains. As previously shown, p38 phosphorylation was decreased during infection by vacuole-restricted bacteria compared to wild-type bacteria [33]. Phosphorylation of p38 upon infection with wild-type *L. monocytogenes* was not significantly affected by XIAP. These data demonstrate that XIAP prolongs JNK activation specifically in response to cytosolic *L. monocytogenes*.

### *L. monocytogenes* induced proinflammatory cytokine expression is enhanced by XIAP

**Figure 5:**
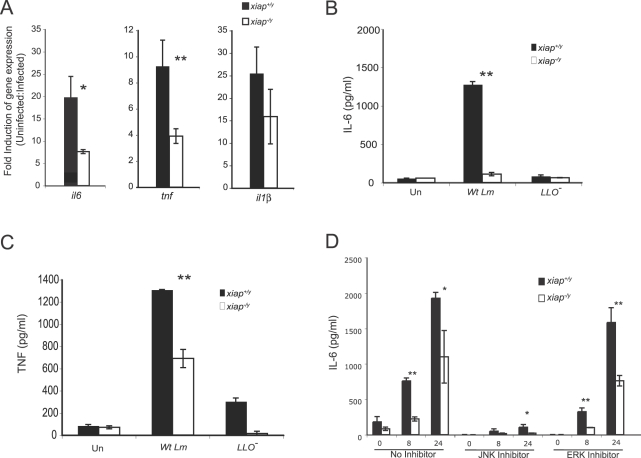
XIAP regulates proinflammatory cytokine expression upon infection with wild-type *L. monocytogenes*. (A) qRT-PCR of genes associated with innate immune activation. BMDM were activated overnight with 10 ng/ml LPS and 10 ng/ml interferon-γ, infected with *L. monocytogenes* for 30 min, and harvested at 3 hpi for RNA isolation and production of cDNA. Fold induction was calculated using the ΔΔC_t_ method, where uninfected samples were compared to infected samples relative to β-actin levels. (B, C) ELISA of IL-6 (B) and TNF (C) secretion from activated BMDM infected with wild-type or LLO^−^ *L. monocytogenes*. Cells were infected with *L. monocytogenes* at an MOI of 10 for 30 min. Supernatants were collected at 8 hpi. (D) ELISA of IL-6 secretion from activated BMDM infected with *L. monocytogenes* and treated with the indicated inhibitors. JNK inhibitor (SP600125) was used at 20 µM, and the ERK inhibitor (U0126) was used at 10 µM. Cells were treated with inhibitors for 1 h, infected at an MOI of 10 for 30 min, and washed with PBS; then, fresh medium with 50 µg/ml gentamicin and the indicated inhibitor was added. Supernatants were collected at 8 and 24 hpi. Error bars represent the SD of macrophages from 3 animals. Results are representative of at least 3 independent experiments. *p≤0.05; **p≤0.005.

Since XIAP modulated JNK and NF-κB signaling in the context of infection, we hypothesized that induction of proinflammatory cytokines through these pathways would also depend on XIAP. Activated macrophages were infected with *L. monocytogenes* for 3 h, and RNA was analyzed by qRT-PCR to determine the expression of a subset of genes involved in innate immunity (Figures 5A and S3). Transcription of *il6*, *tnf*, *il10*, *mip2*, and *kc* was strongly upregulated upon infection in the presence of XIAP, while induction of *ifnb*, *il1b*, *ido*, and *inos* was not significantly altered. To assess if XIAP-dependent gene expression correlated to increased protein production, we compared the secretion of IL-6 and TNF from uninfected and infected activated macrophages (Figure 5B and 5C). Upon infection by wild-type *L. monocytogenes*, IL-6 and TNF secretion was induced to a greater extent in *xiap^+/y^* macrophages than in *xiap^−/y^* macrophages, while infection with the LLO^−^ mutant induced little IL-6 and TNF secretion by either genotype. To determine if JNK activation was required for induction of IL-6 gene expression and secretion in response to wild-type *L. monocytogenes* infection, we treated activated macrophages with the JNK inhibitor SP600125 (Figure 5D). IL-6 secretion by infected macrophages was markedly diminished by JNK inhibition, indicating that JNK activation is required for IL-6 induction by *L. monocytogenes*. Moreover, since LLO^−^ mutant bacteria stimulated robust but temporally limited JNK phosphorylation and little IL-6 secretion, we infer that prolonged JNK activation is necessary for maximal IL-6 production during intracellular infection by *L. monocytogenes*. When *L. monocytogenes* infected cells were treated with an ERK-specific inhibitor, IL-6 secretion was similar to the untreated infected control cells. These results collectively suggest that the presence of XIAP enhances JNK activation in response to cytosolic bacteria, resulting in increased production of proinflammatory cytokines.

### XIAP promotes proinflammatory cytokine expression *in vivo* during *L. monocytogenes* infection

**Figure 6:**
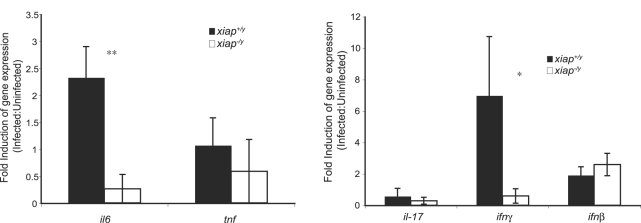
*In vivo L. monocytogenes* infection induces XIAP-dependent pro-inflammatory cytokine expression. qRT-PCR of genes associated with innate immune activation. Mice were infected with *L. monocytogenes*, and splenocytes were harvested at 48 hpi for RNA isolation and production of cDNA. Fold induction was calculated using the ΔΔC_t_ method, where uninfected samples were compared to infected samples relative to β2M levels. *p≤0.05; **p≤0.005.

To determine if XIAP enhanced proinflammatory gene expression *in vivo*, we performed qRT-PCR analysis on splenic RNA. RNA was isolated from splenocytes harvested from uninfected animals or animals infected with *L. monocytogenes* for 48 h (Figure 6). We examined the expression of several proinflammatory cytokines including IL-6, TNF, and IFN-γ, produced during the innate immune response that are critical for clearing *L. monocytogenes* infection [34]–[36]. The expression of *il6* and *ifng* were significantly enhanced in the presence of XIAP during infection, while expression of *tnf* and *ifnb* were not altered. We also examined the expression of *il17*, a cytokine known to enhance expression of *il6*; we observed no reproducible differences in the expression of *il17* [37]. These data confirm the results from our *in vitro* macrophage model; that XIAP promotes the expression of proinflammatory cytokine genes in response to *L. monocytogenes* infection.

### XIAP enables synergy between TLR and NLR signaling

**Figure 7:**
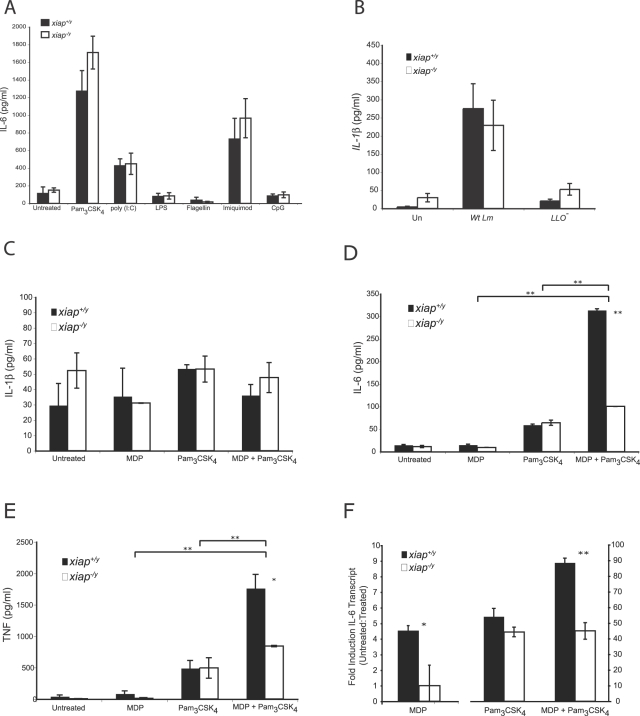
XIAP enables synergistic cytokines responses to TLR and NLR ligands. (A) IL-6 secretion from *xiap^+/y^* and *xiap^−/y^* activated BMDM treated with the indicated TLR ligands as measured by ELISA. Macrophages were activated overnight with 10 ng/ml LPS and 10 ng/ml interferon-γ. Cells were left untreated or were treated for 24 h with Pam_3_CSK_4_ (2 µg/ml), poly (I:C) (10 µg/ml), LPS (10 ng/ml), flagellin (10 ng/ml), imiquimod (5 µg/ml), or CpG DNA (1 µg/ml). Results are representative of at least 3 independent experiments (error bars represent SD). (B) IL-1β from the supernatants of *xiap^+/y^* and *xiap^−/y^* activated BMDM left uninfected or infected with wild-type or LLO^−^ *L. monocytogenes* as measured by ELISA. Supernatants were collected at 8 hpi. Results are representative of 3 independent experiments (error bars represent SEM of cells from 6 animals). (C–E) ELISA of IL-1β (C), IL-6 (D), or TNF (E) secretions from *xiap^+/y^* and *xiap^−/y^* activated BMDM left untreated or treated for 8 h with MDP (10 µg/ml) and/or Pam_3_CSK_4_ (0.5 µg/ml). (F) QRTPCR analysis of IL-6 gene expression at 3 h in *xiap^+/y^* and *xiap^−/y^* activated BMDM treated with MDP (10 µg/ml) and/or Pam_3_CSK_4_ (0.5 µg/ml). The data shown are from the same experiment, but are represented on different graphs to show *y* values more accurately. Data are representative of 3 independent experiments with 3 mice each (error bars represent SD). *p≤0.05; **p≤0.005.

Innate immune signaling mediated by pattern recognition receptors, located on cellular membranes or in the host cytosol, stimulates transcription and secretion of proinflammatory cytokines. We used purified TLR and NLR ligands to better define a role for XIAP in innate immune signaling. Wild-type and XIAP-deficient activated macrophages were treated with TLR ligands, and secretion of IL-6 and TNF was measured after 24 h (Figure 7A and unpublished data). While some PAMPS, such as the lipoprotein Pam_3_CSK_4_, could induce high levels of IL-6 and TNF, we found no XIAP-dependent differences in proinflammatory cytokine induction. These results suggest that XIAP does not contribute to cytokine output in response to TLR stimulation alone.

During a physiological infection, intracellular pathogens activate both extracellular and cytosolic innate immune pathways resulting in a coordinated immune response [27]. One well-characterized consequence of microbial sensing by cytosolic NLR proteins is activation of caspase-1, which cleaves pro-IL-1β into its mature form [38]. Since XIAP can regulate the activity of some caspases, we tested whether XIAP contributed to IL-1β production, measured by ELISA, as an indicator of caspase-1 activation (Figure 7B). Consistent with previous reports, IL-1β production was induced by cytosolic *L. monocytogenes*, but was not dependent upon XIAP [39]. We next examined the activation of NLR signaling using MDP, a ligand for NOD2 (Figure 7C–7E). No differences in cytokine secretion were observed by treatment with MDP alone, however, during a physiological infection bacteria likely present both TLR and NLR ligands to an infected host cell. PAMPs contained by *L. monocytogenes* include lipoprotein, muramyldipeptide, bacterial DNA and flagellin [27]. To determine if XIAP enhanced synergy between TLRs and NLRs, we examined IL-6, TNF and IL-1β secretion from *xiap^+/y^* and *xiap^−/y^* activated macrophages in response to the lipopeptide Pam_3_CSK_4_, the NOD2 ligand MDP, or both (Figure 7C–7E). When Pam_3_CSK_4_ and MDP were used in combination, we saw a substantial increase in IL-6 and TNF secretion by *xiap^+/y^* but not *xiap^−/y^* activated macrophages. We did not see any XIAP-dependent enhancement of IL-1β secretion in response to Pam_3_CSK_4_ and MDP in combination. To better deconstruct how XIAP might participate in integrating TLR and NLR signaling, we analyzed transcription of the *il6* gene from *xiap^+/y^* and *xiap^−/y^* activated macrophages treated with MDP, Pam_3_CSK_4_, or both ligands (Figure 7F). Pam_3_CSK_4_ induced expression of the *il6* gene in an XIAP-independent manner. Upon treatment with MDP, *xiap^+/y^* but not *xiap^−/y^* macrophages, responded by upregulating *il6* transcript levels approximately 5-fold. When macrophages were treated with both ligands, *xiap^+/y^* macrophages exhibited enhanced expression of *il6* compared to treatment of Pam_3_CSK_4_ alone, but *xiap^−/y^* macrophages did not. These results demonstrate that XIAP promotes synergy between the TLR and NLR pathways, resulting in increased production of pro-inflammatory cytokines.

## Discussion

Here we show that XIAP can regulate innate immunity to the bacterial pathogen, *L. monocytogenes* by modulating JNK and NF-κB signaling, resulting in enhanced cytokine production. We found little evidence to suggest that XIAP regulated apoptosis of bacterially infected cells *in vitro* or *in vivo*, but instead found that XIAP promoted synergistic inflammatory cytokine expression induced by extracellular and cytosolic innate immune signaling upon bacterial infection of activated macrophages. Specifically, XIAP amplified the cytosolic response to MDP or wild-type *L. monocytogenes*. These data identify XIAP as a regulator of cytosolic innate immune signaling. Notably, another IAP family member NAIP5 was found to mediate caspase-1 activation in response to cytosolic bacterial flagellin [40]–[42]. NAIP5 function in innate immunity could be attributed to the atypical domain structure of this IAP protein that exhibits similarities to the NLR family of cytosolic sensors [43]. However, these data taken together with our results lead us to speculate that regulation of innate immune signaling is an important role of mammalian IAPs.

The IAP family appears to play multiple roles in mammalian biology, including protecting cells from apoptotic stimuli, regulating the cell cycle and modulating innate immune signaling. As a whole, these studies are consistent with genetic evidence in *Drosophila* demonstrating that dIAP1 primarily protects insect cells from programmed cell death, while dIAP2 is required for anti-microbial function of the Imd pathway [4]–[7]. The Imd pathway in *Drosophila* is activated by peptidoglycan recognition proteins (PGRPs), while functionally analogous innate immune sensing of peptidoglycan in mammalian cells occurs in the cytosol by NOD1, NOD2, and NALP3 [44]. The Imd protein in *Drosophila* shares sequence homology with the mammalian RIP proteins, and a mammalian paralog, RIP2, is an essential signaling adaptor for the cytosolic peptidoglycan sensors, NOD1 and NOD2 [8], [45]–[47]. Thus, the Imd/RIP innate immune signaling module appears to have been co-opted for mammalian cytosolic surveillance for peptidoglycan. Genetic epistasis experiments in *Drosophila* place dIAP2 in parallel to TAK1 upstream of JNK and NF-κB signaling pathways [4]. Similarly, in mammalian cells, XIAP can modulate JNK and NF-κB signaling through TAK1 in endothelial cells and fibroblasts [13],[48]. Activation of either NOD1 or NOD2 activates TAK1, leading us to hypothesize that during bacterial infection, XIAP may facilitate this key association, linking cytosolic sensors to downstream signaling mediators [49],[50].

During infection, microbial pathogens present multiple PAMPs recognized by the innate immune system, eliciting a coordinated protective response. This concept is illustrated by the paradigm of IL-1β processing, where TLRs mediate transcription of pro-IL-1β; however, cleavage and secretion are dependent upon activation of the caspase-1 inflammasome by cytosolic PAMPs [51]. However, IL-1β deficient mice are as resistant to *L. monocytogenes* infection as wild-type mice, suggesting that other inflammatory cytokines mediate innate immune control of this infection [52]. In contrast, IL-6-, TNF- and IFNγ-deficient mice are more susceptible to *L. monocytogenes* infection at 48 hpi than wild-type mice, demonstrating a requirement for IL-6, TNF, and IFNγ in protection from this particular pathogen [34]–[36],[53],[54]. IFNγ is largely produced by innate immune effector cells other than macrophages, thus our observation that *ifng* transcription is decreased in the spleens of *L. monocytogenes*-infected XIAP mutant mice must be due to either a XIAP-dependent cell autonomous defect in a different cell type or a non-autonomous defect in an IFNγ producing cell resulting from a defect in macrophages [55]. Since XIAP is expressed in many different tissues, it is reasonable to suppose that XIAP may have pleiotropic effects in the innate immune response to *L. monocytogenes* [56]. However, macrophages are primary producers of IL-6 and TNF, and notably, NOD2 signaling is known to stimulate production of IL-6 and TNF [45],[57]. The deficit in IL-6 and TNF production we observed in infected *xiap^−/y^* activated macrophages, and the defect in gene expression *in vivo* likely contributes to the enhanced susceptibility of XIAP-deficient animals to *L. monocytogenes* infection. Recent reports indicate that macrophages treated with LPS become tolerized to re-stimulation with TLR ligands [58],[59]. Additionally, when macrophages are tolerized by LPS, the role of NOD1 and NOD2 in cytosolic surveillance becomes more critical during infection [60]. In our model, macrophages are activated with LPS and IFNγ prior to infection. When activated macrophages are infected with *L. monocytogenes*, the induction of proinflammatory cytokines is XIAP-dependent, indicating that XIAP plays a more critical role in regulating the innate immune response to cytosolic pathogens in macrophages where the TLR pathway may be tolerized and an inflammatory gene expression program initiated. We use these data to integrate XIAP into a cytosolic surveillance model whereby upon recognition of microbial ligands in the cytosol by innate immune sensors such as NOD2, XIAP enhances association and function of signal transducers such as TAK1 and JNK [13],[18]. Recruitment of signaling molecules by XIAP upon NLR stimulation would potentiate signaling pathways activated by TLRs, leading to maximal proinflammatory cytokine production.

Apoptotic and microbial stimuli activate similar signaling pathways, but may lead to different outcomes. Macrophages as innate immune effector cells can control microbial infection by secreting cytokines and other pro-inflammatory molecules or by carrying out programmed cell death [61]. It has been hypothesized that when macrophages receive a strong inflammatory stimulus, they undergo apoptosis rather than secreting cytokines as a means of protecting the host [40],[62],[63]. Although previous data implicated XIAP in modulating apoptosis, our data demonstrate that XIAP also has an important role in proinflammatory cytokine production. However, we suggest that these two functions for XIAP may not be completely distinct, as the outcome of XIAP-dependent modulation of JNK and NF- κB pathways may depend on the quality and intensity of the stimulus [31]. Additionally, the ability of XIAP to regulate innate immunity is likely cell type and context dependent, as we did not see reproducible XIAP-dependent transcriptional regulation in unactivated macrophages. Future studies will determine which aspects of XIAP function contribute to immune signaling and elucidate the complex role of XIAP in the mammalian immune response.

## Materials and Methods

### Animals, bacterial strains, and infections

Mice deficient in XIAP (accession #U88990) were generated on a 129/Sv×129/SvJ background as previously described [20]. The XIAP-deficient mice were backcrossed onto the C57Bl/6 background for more than 10 generations. Six- to 12-week-old male XIAP-deficient mice or wild-type littermate controls were used for infection experiments. All animals received humane care as outlined by the Guide for the Care and Use of Laboratory Animals (University of Michigan Committee on Use and Care of Animals). For cell culture infections, *Listeria monocytogenes* strains 10403S (wild-type) and *hly* ^−^ (LLO^−^) were inoculated into liquid brain-heart infusion (BHI) broth and incubated at 30°C overnight without shaking[64]. Prior to infection, *L. monocytogenes* cultures were washed and resuspended in PBS. HKLM was prepared by incubating bacteria at 70°C for 1 h. For animal infections, *L. monocytogenes* was grown to log-phase in BHI and aliquots were stored at −70°C. For each experiment, a vial was back-diluted and allowed to grow to OD_600_ 0.5. The bacteria were washed in PBS and diluted before injection. Mice were injected intraperitoneally with 5×10^5^ *L. monocytogenes* equivalent to 0.5 LD_50_ for infection by the intraperitoneal route in C57Bl/6 mice [65]. The number of viable bacteria in the inoculum and organ homogenates was determined by plating 10-fold serial dilutions on Luria broth (LB) agar plates. For evaluation of survival, animals were infected with 1×10^5^ or 5×10^5^ *L. monocytogenes*, and observed every 24 h post-infection. For histology, the spleen and liver from infected mice were harvested at 48 hpi and fixed in 10% neutral buffered formalin. Paraffin sections were prepared and stained with ApopTag by the Cancer Center Research Histology and Immunoperoxidase Lab at the University of Michigan.

### BMDM culture

Bone marrow macrophages were differentiated in DMEM supplemented with 20% heat inactivated FBS, 2 mM L-glutamine, 1 mM sodium pyruvate, 0.1% β-mercaptoethanol, and 30% L929 conditioned medium. Bone marrow cells were cultured at an initial density of 10^7^ cells per 150 mm non-tissue culture treated dish for 6 d, with fresh medium added at day 3. Cells were harvested with cold PBS without calcium and magnesium. BMDM were activated overnight in 10 ng/ml LPS (Sigma #L6143) and 10 ng/ml (100 U/ml) interferon-γ (Peprotech #315-05). Activated macrophages were infected with *L. monocytogenes* at an MOI of 10, such that bacteria were observed in the cytosol in approximately 99% of the macrophages. Peritoneal macrophages were harvested by peritoneal lavage. Cells were pooled from two mice prior to plating. For *L. monocytogenes* growth curves, cells were plated on coverslips at a density of 1.7×10^5^ cells/ml in 24-well plates. Macrophages were infected with *L. monocytogenes* for 0.5 h, washed 3 times with PBS, followed by addition of fresh medium with 50 µg/ml gentamicin. At each time point, 3 coverslips were lysed in water and plated on LB agar plates for to determine CFU. IL-6 (R&D Systems), IL-1β (R&D Systems), and TNF (University of Michigan Cellular Immunology Core) in the culture medium were measured by ELISA. Where indicated, cells were treated for 30 min with TLR ligands as follows: MDP 10 µg/ml (Bachem #4009623), Pam_3_CSK_4_ 2 µg/ml (Invivogen #tlrl-pms), poly (I:C) 10 µg/ml, LPS 10 ng/ml (Sigma #L6143), flagellin 10 ng/ml (Invivogen #tlrl-flic), imiquimod 5 µg/ml (Invivogen #tlrl-imq), CpG DNA 1 µg/ml (IDT CpG F [5′-TCCATGACGTTCCTGACGTT], CpG R [5′-AACGTCAGGAACGTCATGGA]). At 8 and 24 h post-treatment, supernatants were harvested for measurement of cytokines by ELISA. Inhibition experiments were conducted as described above, except cells were treated with 20 µM JNK inhibitor, SP600125 (Sigma #S5567), or 10 µM ERK inhibitor U0126 (Cell Signaling #9903) for 1 h prior to infection. For nuclear and cytoplasmic fractionation, cells were lysed in NP-40 lysis buffer (50 mM Tris pH 8.5 mM EDTA pH 8, 150 mM NaCl, 0.05% NP-40 [Igepal], EDTA-free protease inhibitor cocktail [Roche]). Nuclei were pelleted by centrifugation at 1,000 rpm for 5 min; the cytosolic fraction was further clarified by centrifugation at 14,000 rpm for 10 min. Nuclei were washed and either resuspended in 2× SDS-PAGE lysis buffer for immunoblot or lysed for NF-κB ELISA by resuspension in nuclear lysis buffer (20 mM HEPES pH 7.9, 400 mM NaCl, 1 mM EDTA, 10% glycerol, 0.1 mM DTT, EDTA-free protease inhibitor cocktail [Roche]) and incubated at 4°C for 30 min. Nuclei were flash frozen and used for NF-κB p65 ELISA analysis (Stressgen EKS-446).

### Apoptosis assays

BMDM were plated and activated overnight in 10 ng/ml LPS and 10 ng/ml interferon-γ. Cells were infected for 30 min at an MOI of 10, bacteria were removed by 3 washes with PBS, and fresh medium containing 50 µg/ml gentamicin added. At 3 hpi, the medium was removed and spun to collect any non-adherent cells; the remaining cells were removed from the dish by incubating with ice-cold PBS without calcium and magnesium for 20 min at 4°C. Cells were stained with Annexin V and propidium iodide according to the manufacturer's protocol (BD Biosciences #556420).

### Flow cytometry

Splenocytes were harvested from uninfected or *L. monocytogenes* infected mice. BMDM were harvested from plates with ice cold PBS without Ca^+^ or Mg^+^. Cells were blocked with F_c_ block (BD Pharmingen 553142) for 15 min on ice. Cells were incubated in staining buffer (PBS, 10% FBS) with the indicated antibodies for 20 min on ice, followed by 3 washes in staining buffer. When necessary cells were incubated with secondary antibodies in staining buffer on ice for 20 min, and washed 3 times in staining buffer. Flow cytometric acquisition was performed on a FACSCanto. The data was analyzed using FlowJo software. The following antibodies were used: from BD Pharmingen; B220-PE (553089), NK1.1-biotin (553163), CD69-PE (553237); from Southern Biotech CD3 (1530-02), Streptavidin-APC (7100-11L).

### Immunoblot analysis

Whole cell lysates were generated by adding 2× SDS-PAGE sample buffer directly to cell monolayers. Protein samples were separated by SDS-PAGE and transferred to PVDF. Blots were blocked in 5% BSA, incubated with primary antibodies, followed by a horseradish peroxidase conjugated secondary antibody. The following antibodies were used: β-actin (Sigma #A5441), NF-κB p50 (Santa Cruz Biotechnology #8414), USF-1 (Santa Cruz Biotechnology #8983), Phospho-JNK (Cell Signaling 9251), JNK1 (Santa Cruz Biotechnology #571), Phospho-p38 kit (Cell Signaling 9210), Phospho-c-jun (Santa Cruz Biotechnology #822), Phospho-ERK (Cell Signaling 4377), ERK-1 (Santa Cruz Biotechnology #94), goat anti Rabbit IgG-HRP (MP Biomedical #67438), goat anti-mouse IgG-HRP (MP Biomedical #67429).

### RNA isolation and quantitative RT-PCR analysis

For RT-PCR, total RNA was harvested from infected or treated cells at 3 hpi with the RNeasy Mini Kit (Qiagen). The RNA was used in a reverse transcriptase (RT) reaction with Moloney murine leukemia virus (MMLV) RT (Invitrogen). cDNA obtained from the RT reaction was used for qRT-PCR amplification and quantitation by SYBR Green (Stratagene MX3000p). Data was analyzed using the ΔΔC_t_ method (ΔΔC_t_ = 2^(ΔCt sample−ΔCt normalizer)^) with *β-actin* used as a normalizer for *in vitro* experiments and *gapdh* used as a normalizer for *in vivo* experiments. Sequences for qRT-PCR primers are described in Table S1.

### Statistical analysis

A two-tailed *t*-test was used for statistical analysis; *p* values of ≤0.05 were considered significant, while *p*≤0.001 were considered highly significant.

## Supporting Information

Figure S1 XIAP does not appear to modulate apoptosis during *L. monocytogenes* infection. (A) Apoptosis in activated BMDM infected with *L. monocytogenes* at 6, 8, and 24 hpi, as determined by flow cytometric analysis of Annexin V and propidium iodide staining. Results are representative of at least 3 independent experiments (error bars represent SD of cells from 3 mice). (B) Apoptosis of T cells (CD3+) and B cells (B220+) from uninfected and *L. monocytogenes* infected splenocytes, as determined by Annexin V and PI staining. Results are representative of at least 3 independent experiments (n = 9 animals/genotype).
(3.85 MB EPS)

Figure S2 Quantitation of MAP kinase phosphorylation by immunoblot. Blots from Figure 4 were quantitated based upon band density, as determined by ImageJ software. Band intensities were compared to the first sample of the blot, whose value was arbitrarily set to 1. (A) P-JNK blot quantitation. (B) P-cjun quantitation. (C) P-38 blot quantitation. (D) P-ERK blot quantitation.
(444 KB EPS)

Figure S3 XIAP regulates induction of multiple proinflammatory response genes. QRT-PCR of genes associated with innate immune activation. BMDM were activated overnight with 10 ng/ml LPS and 10 ng/ml interferon-γ, followed by with *L. monocytogenes* for 30 min, and harvest at 3 hpi. RNA was isolated and used to prepare cDNA for qRT-PCR. Fold induction was calculated using the ΔΔCt method, where uninfected samples were compared to infected samples relative to β-actin levels (error bars represent SD). *p≤0.05; **p≤0.005.
(2 MB EPS)

Table S1 Primers used in this study
(43 KB DOC)
