# Supplementary material for: XIAP Regulates Cytosol-Specific Innate Immunity to Listeria Infection
Source: PLoS Pathog. 2008 Aug 29;4(8):e1000142. doi: 10.1371/journal.ppat.1000142 (PMC2516935; doi:10.1371/journal.ppat.1000142)
Supplement: Table S1 — Primers used in this study (43 KB DOC) [file ppat.1000142.s004.doc]

**Supporting Table 1. Primers used in this study.**

| **Gene** | **Forward Primer Sequence** | **Reverse Primer Sequence** | **Accession #** |
| --- | --- | --- | --- |
| * β-actin* | 5’-CGCATCCTCTTCCTCCCT | 5’-GGATGCCACAGGATTCCA | NM_007393 |
| *ifnb* | 5’-AAGCAGCTCCAGCTCCAA | 5’-TTGGATGGCAAAGGCAGT | NM_008337 |
| *il6* | 5’-TTGGGACTGATGCTGGTGACA | 5’-ATGGTACTCCAGAAGACCAGA | J03783 |
| *il10* | 5’-GGGTTGCCAAGCCTTATCGGAAAT | 5’-TGGCCTTGTAGACACCTTGGTCTT | NM_010548 |
| *ido* | 5’-GCAGCTTCTCCTGCAATCAAAGCA | 5’-ATACAGCAGACCTTCTGGCAGCTT | BC049931 |
| *inos* | 5’-TCTTGACGCTCGGAACTGTAGCA | 5’-TAGGTCGATGCACAACTGGGTGAA | NM_010927 |
| *mip2* | 5’-AAAGTTTGCCTTGACCCTGAA | 5’-TCTTTGGTTCTTCCGTTGAGG | X53798 |
| *kc* | 5’-ACCCAAACCGAAGTCATAGCC | 5’-AGTGTTGTCAGAAGCCAGCGT | J04596 |
| *tnf* | 5’-ACGGCATGGATCTCAAAGACAACC | 5’-TGAGATAGCAAATCGGCTGACGGTT | M11731 |
| *ifng* | 5’-AGGCCATCAGCAACAACATAAGCG | 5’-GGGTTGTTGACCTCAAACTTGGCA | NM_008337 |
| *il17* | 5’-AACATGAGTCCAGGGAGAGCTTCA | 5’-AGTGTTTGGACACGCTGAGCTTTG | NM_010552 |
| *b2m* | 5’-ACCGGCCTGTATGCTATCCAGAAA | 5’-ATTTCAATGTGAGGCGGGTGGAAC | BC085164 |
| *il1b* | 5’-AAGAGCTTCAGGCAGGCAGTATCA | 5’-TGCAGCTGTCTAATGGGAACGTCA | M15131 |
